# Emotional abuse towards children by schoolteachers in Aden Governorate, Yemen: A cross-sectional study

**DOI:** 10.1186/1471-2458-12-647

**Published:** 2012-08-13

**Authors:** Amal S S Ba- Saddik, Abdullah S Hattab

**Affiliations:** 1 Department of Behavioral Sciences, Faculty of Medicine & Health Sciences, Aden University, P.O. Box 6165 (Khormaksar), Aden, Yemen; 2 Department of Social Medicine &Public Health, Faculty of Medicine &Health Sciences, Aden University, P.O. Box 6165 (Khormaksar), Aden, Yemen

**Keywords:** Emotional abuse, Teachers, Schoolchildren, Aden

## Abstract

**Background:**

Emotional abuse is central to other forms of abuse. The primary objective of this paper was to estimate the prevalence of emotional abuse among pupils in basic education schools and the risk factors associated with it in Aden governorate, Yemen.

**Methods:**

Four districts were randomly selected from across the governorate of Aden, 2 schools were selected at random in each district, and then 1066 pupils were randomly selected from the 8 schools. An anonymous self-administered questionnaire was used for data collection. Data were analyzed using Statistical Package for Social Sciences ver.15. Mean, standard deviation and chi square were used for descriptive statistics. Univariate and Multivariate logistic regression analysis was used to examine the associations between emotional abuse with pupils/parents characteristics.

**Results:**

Pupils reported high rates of emotional abuse 55.2% at least once in their school lifetime. Male pupils had higher prevalence of emotional abuse 72.6% than females 26.1%. Teachers constituted the highest proportion of perpetrators 45.6%. Odds Ratio (95% confidence interval) showed statistically significant association between emotional abuse and pupils' gender, family type and father education: 9.94 (7.19-13.74), 1.40 (1.02-1.91), .58 (.39-.86) respectively.

**Conclusion:**

Emotional child abuse was highly prevalent in pupils in basic school education. Pupils' gender, family type and father education were the main risk factors associated with emotional abuse.

## Background

Emotional abuse may be the most prevalent type of child abuse; however, it is also the most hidden, under-reported, and least studied type of abuse [1,2]. Literature on emotional abuse is limited, which could be attributed to the fact that it is the most difficult form of abuse to research, because of lack of a consistent definition, detect, assess, and substantiate [2].

A growing body of research highlights the harmful effects of emotional maltreatment in children. As such, victims experience difficulties in terms of physical health and neurophysiological, emotional, behavioral, and cognitive development [3]. Also, it is significantly related to subsequent delinquent behavior and academic difficulties in early adolescence [4].

Many psychologists assert that emotional abuse is the most devastating form of child abuse, because of its traumatic effects in the development of school pupils [2,5,6], and it underlies all types of child abuse as a perpetrator of emotional abuse can abuse many victims at one particular moment [7].

To date, studies on pupils' abuse by teachers are relatively limited and those that exist mainly focus on corporal punishment [8-10]. Nevertheless, high prevalence of emotional child abuse was reported from India, United States, Zimbabwe, Nigeria and Cyprus [11-15]. The study from India found that 47.9% of surveyed boys and 52.1% of girls had experienced emotional abuse in schools [11].

It is important to explore the issue of pupils abused by educators in educational settings in the Eastern Mediterranean regions, since only two studies [16,17] have indicated that pupils' abuse in schools was enormous. In Bahrain, Al-Mahrous in 1997 found that emotional abuse was very frequently reported by girls 78% [16], while a study from Iran showed that emotional abuse among schoolgirls was 49.8% [17].

In Yemen, documented studies on child abuse are very scarce. Nevertheless, literature search revealed two important studies that addressed the problem of child abuse in family setting [18,19]. The third one [20] was more comprehensive covering the problem of child abuse in family, community and school setting and showed that 42.3% of the school pupils experienced humiliating as an undesirable emotional behavior by their teachers.

This study aimed to assess the prevalence of emotional abuse among schoolchildren in Aden schools and associated factors. Also, we expect that it will contribute to better understanding of emotional abuse in school setting in a developing country.

## Methods

### Study design and setting

This study is a part of a more comprehensive cross-sectional survey of different types of abuse among schoolchildren of basic education in Aden Governorate in Yemen that was conducted during the school year 2009–2010.

### Study population

Pupils of grades 7, 8 and 9 were targeted in the survey. Children, at the age of 12–17 years, usually are capable of perceiving what is and is not abuse within the school context, they also could provide reliable information, and are capable of answering the questionnaire [10,21].

### Sample size

The sample size was calculated using the assumed proportion of 0.5 in order to obtain the maximum possible sample size, with a level of confidence 95%, and 0.03, as maximum allowable error. Accordingly, the calculated sample size was 1066 pupils who were proportionally distributed according to pupils' gender (667 males and 399 females).

### Sampling method

A multi-stage stratified random sampling was performed. In the first stage, four districts were randomly selected. In the second stage, two schools from each district also were randomly selected. In the third stage, the sample size (1066) was proportionally distributed according to the proportion of students in the selected grades for each school by gender. The sample frame for this study was the pupils' list in the selected grades of the schools incorporated in this study. Systematic random sampling was applied to select the number of pupils assigned for each grade in the selected schools.

### Instrument

An anonymous self-administrated questionnaire adapted from the Arabic version of Child Abuse Screening Tool Children's Institutional Version [21], was used for data collection.

The first part of the instrument covers questions about pupil's variables: gender, age, school grade, residence, in addition to parent's socio-demographic variables: family type, parent's education and parent's marital status.

The second part includes 10 items including acts such as humiliating, shouting, calling names, embarrassing them of being orphan, poor, having health problems, threatening with giving them bad marks, or expelling from school, isolating them from other children and destroying their belongings.

Experts in child abuse from Yemeni Universities judged the questionnaire, to find out if it is socially acceptable. Accordingly, some items were rephrased, and other items were dropped out. The final modified version of the questionnaire used in this study included the following questions:

Have you ever been exposed to any of these acts at school?

Humiliating, shouting, calling names, embarrassing of being orphan, embarrassing of being poor, threatening with bad marks, threatening to expelling from school, embarrassing for having health problems, isolating from children, destroying belongings. Pupils who answered with "Yes" were asked to report the frequency of the abuse acts by indicating 1-2 times, 3-4 times, ≥ 5 times. In addition they were asked to mention who did it: a teacher or a school administrator? [21].

### Pilot study

A pilot study was conducted among 60 pupils (30 males and 30 females) from two schools not included in the main study, to ensure that the questionnaire items were clear, understandable and culturally acceptable. Chronbach alpha was used to test the internal consistency reliability, which was found to be 0.78.

We explained the questionnaire in detail to the pupils, and asked them to answer '*yes'* or *'no*' to each item and how many times they have experienced abusive acts during scholastic years. Those who responded affirmatively were asked to identify the perpetrators: *teacher* or *school administrative staff*. The rate of physical abuse was calculated by recoding the acts into dichotomous categories, with 0 = never and 1 = once or more [22]. A 4- point scale (0 = none; 1 = 1-2 times; 2 = 3–4 times; 3 = 5 times and more) was used to indicate how often they had experienced each abuse act [23].

### Operational definition

*Emotional abuse* in this study refers to pupils' reports of any undesirable or unpleasant emotional act inflicted on them by teachers or other school administrators (school principal, vice principal or other workers) which could potentially make them feel embarrassed while in school. Emotionally abused pupils were defined as those who answered positively to one or more of the emotional abuse acts.

### Ethical considerations

1. The research protocol was approved by the *Committee of Research and Postgraduate Studies* in the Faculty of Medicine and Health Sciences, Aden University. Several levels of permission were granted before the study could proceed, including official approval from the authority of Aden Education Office. In addition, permission was sought from the Districts Directors of Education, as well as from school principals.

2. A written informed consent was sent to the pupils' parents describing the nature of the study, its importance, and its objectives. The consent also stated that the data's confidentiality would be assured, that the participation in the study was voluntary and those who refused participation will not lose any rights or privilege. Parents were asked to put their signature if they agree to have their child participate in the survey. The response rate was 85% of the targeted parents. For those who declined to consent, equal number was substituted following the same methodology.

3. The pupils' informed assent was obtained orally during which detailed explanation of the objectives and the importance of the research was provided. Potential participants were assured that all information obtained will be handled confidentially. Pupils were informed that they have the right to decline answering any question and/or to withdraw from the study at any time. All pupils whose parents gave consent to their participation have assented to participate in the study.

### Data analysis

The Statistical Package for Social Science -SPSS – version 15 was used for data analysis. Quantitative variables were normally distributed after testing for normality using Kolmogorove-Smirnov test. Percentage was calculated as summary measure for the qualitative variables. Arithmetic mean, standard deviation and chi square test were used for the descriptive statistics. The statistical significant level was set at p-value < 0.05.

Binary logistic regressions were conducted to describe the bivariate association between emotional abuse and the pupils and parents variables. Multivariate analysis was performed to identify risk factors associated with emotional abuse. The results were discussed in terms of the odds ratio (OR) with 95% Confidence Interval (CI). The OR of the reference category is equal to "1". If an OR is greater than "1" this indicates an increase likelihood of the event (emotional abuse in this research) occurrence, while an OR less than "1" indicates a decreased likelihood of its occurrence.

## Results

### Socio demographic characteristics of the study population

Table 1 shows the socioeconomic characteristics of the study population. Male pupils constituted the highest proportion 62.6%. The mean age was 14.03 ± 1.13 years. Most of the pupils lived in nuclear families. More than 50% of the mothers were illiterates or could only read and write.

**Table 1:** Distribution of pupils and their families by socio- demographic characteristics

**Pupils characteristicsllr**	**No.**	**%**
**Gender**		
Male	667	62.6
Female	399	37.4
**Age group (years)**		
12-13	363	34.0
14-15	618	58.0
16-17	85	8.0
Mean age = 14.03 ± 1.13 years		
**School grade**		
Grade 7	322	30.2
Grade 8	366	34.3
Grade 9	378	35.5
**Family socio-demographic characteristics**		
**Family type**		
Nuclear	747	70.1
Extended	319	29.9
**Parents marital status**		
Married	952	89.3
Separated	21	2.0
Divorced	33	3.1
Widow	60	5.6
**Father education**		
Illiterate	74	6.9
Read and write	281	26.4
Basic	103	9.7
Secondary	202	18.9
University	406	38.1
**Mother education**		
Illiterate	264	24.8
Read and write	324	30.4
Basic	177	16.6
Secondary	162	15.2
University	139	13.0

### Prevalence of emotional abuse

The study sample included 1066 pupils: 588 of them reported one or more emotional abuse acts, before the age of 18 years. The prevalence of emotional abuse in the surveyed sample was 55.2% (95% CI: 52.1 - 58.2). Teachers occupied the higher proportion of being responsible for emotional abuse 45.6%, followed distantly by the administrative staff 5.0%. As summarized in Table 2. The most common emotional abuse act reported by pupils was *shouting* 48.1%, the next most common emotional abuse acts were *calling names* 36.1% and *threatening to give bad marks* 31.9%. The lowest emotional abuse acts the pupils experienced was being embarrassed by teachers and school administrators for *being an orphan* 1.7% and 2.0% reported being threatened that their belongings will be destroyed. However, it is important to note that there are gender statistically significant differences: Males had higher prevalence rates than females on almost all of the acts of abuse.

**Table 2:** Distribution of emotional abuse acts by gender

**Emotional abuse acts**	**Gender**						***P*-value**
	**Male**		**Female**		**Total**		
	**(n = 667)**		**(n = 399)**		**1066**		
Humiliating	221	33.1	82	20.5	303	28.4	0.000
Shouting	351	52.6	162	40.6	513	48.1	0.022
Calling names	290	43.5	95	23.8	385	36.1	0.000
Embarrassed of being orphan	17	2.54	1	0.3	18	1.7	0.005
Embarrass of being poor	51	7.6	6	1.5	57	5.3	0.000
Threaten with bad marks	242	36.3	98	24.6	340	31.9	0.003
Threaten to drop out school	268	40.2	65	16.3	333	31.2	0.000
Embarrass by having health problems	38	5.7	14	3.5	52	4.8	0.125
Isolated from children	59	8.8	15	3.8	74	6.9	0.002
Destroying belongings	19	2.8	2	0.5	21	2.0	0.008

The frequency of emotional abuse acts is presented in Table 3. Abuse acts that were experienced 1–2 times were shouting 20.7%, threaten to drop out school 16.9% and calling names 15.0%. On the other hand, those acts that were experienced 3–4 times were shouting 12.1%, calling names 8.3%, and threaten with bad marks 8.2%. While those acts that were experienced five times or more were shouting 15.3%, calling names 12.8% and humiliating 9.6%.

**Table 3:** frequency of emotional abuse acts

**Emotional abuse acts**	**Never**		**1-2 times**		**3-4 times**		**≥ 5 times**	
	**No.**	**%**	**No.**	**%**	**No.**	**%**	**No.**	**%**
Humiliating	763	71.6	134	12.6	66	6.2	103	9.6
Shouting	553	51.9	221	20.7	129	12.1	163	15.3
Calling names	681	63.9	160	15.0	88	8.3	137	12.8
Embarrassed of being orphan	1048	98.3	12	1.1	3	0.3	3	0.3
Embarrass of being poor	1009	94.7	28	2.6	14	1.3	15	1.4
Threaten with bad marks	726	68.1	156	14.6	87	8.2	97	9.1
Threaten to drop out school	733	68.8	180	16.9	68	6.4	85	7.9
Embarrass by having health problems	1014	95.1	26	2.4	17	1.6	9	0.8
Isolated from children	992	93.1	32	3.0	27	2.5	15	1.4
Destroying belongings	1045	98.0	8	0.8	2	0.2	11	1.0

### Association analysis

#### Univariate analysis

Table 4 shows the association of the pupils' socio-demographic factors with emotional abuse. Gender and age group have a significant association with emotional abuse. Male had a significantly higher odd of experiencing emotional abuse (OR = 7.50; CI: 5.66 - 9.93) than females. With respect to the age group, emotional abuse significantly increases at the age group 16–17 years (OR = 1.91; 95%CI: 1.17-3.11). The univariate analysis does not show any significant association between school grade and emotional abuse.

**Table 4:** Univariate analysis for pupils' variables and emotional abuse

**Variables**	**Emotional abuse**				
	**No**	**%**	**OR**	**(95 % CI)**	***P*-value**
**Gender**					
Female*	104	26.1	1.00		
Male	484	72.6	7.50	5.66 - 9.93	.000
**Age group**					
12-13*	173	47.7	1.00		
14-15	361	58.4	1.54	1.18 –2.00	.001
16-17	54	63.5	1.91	1.17 – 3.11	.009
**School grade**					
Grade 7*	171	53.1	1.00		
Grade 8	212	57.9	1.21	.89 – 1.64	.205
Grade 9	205	54.2	1.04	.77– 1.41	.766

* Reference category.

Table 5 illustrates that pupils living in extended families were more likely to experience emotional abuse (OR = 1.55; 95%CI: 1.18-2.03). Concerning parents' marital status, only pupils with divorced parents were significantly more likely to experienced emotional abuse (OR = 2.20; CI: 1.01-4.79). On the other hand, the high education levels of fathers play a protective role against pupils' emotional abuse (OR = .64; 95%CI: .46-.90).

**Table 5:** Univariate analysis for parent's variables and emotional abuse

**Variables**	**Emotional abuse**				
	**No**	**%**	**OR**	**(95 % CI)**	***P*-value**
**Family type**					
Nuclear*	388	51.9	1.00		
Extended	200	62.7	1.55	1.18- 2.03	.001
**Father education**					
Illiterate*	46	62.2	1.00		
Read/write	162	57.7	1.28	.77-2.13	.338
Basic	61	59.2	1.06	.78-1.44	.698
Secondary	91	45.0	1.13	.73-1.75	.575
University	228	56.2	.64	.45-.89	.010
**Mother education**					
Illiterate*	151	57.2	1.00		
Read/write	190	58.6	1.28	.84-1.93	.241
Basic	95	53.7	1.35	.91-2.02	.133
Secondary	81	50.0	1.11	.71-1.73	.647
University	71	51.1	.95	.60-1.50	.852
**Parents Marital Status**					
Married*	521	54.7	1.00		
Separated	13	61.9	1.34	.55-3.27	.515
Divorced	24	72.7	2.20	1.01-4.79	.046
Widow	30	50.0	.82	.49-1.39	.476

* Reference category.

#### Multivariate analysis of predictors of emotional abuse

All the variables that were significant in the univariate models with a p value < 0.05 were entered into the backward logistic regression. The final multivariate logistic regression models included all variables retaining significant after adjusting for each other. As can be seen in Table 6 the significant predictors were male gender, family type and father education. Male pupils had about ten times greater risk of experiencing emotional abuse (OR = 9.94; CI: 7.19-13.74) than female. On the other hand, a protective association was found among pupils belonging to highly educated fathers (OR = .58; CI: .39-.86).

**Table 6:** Multivariate analysis for pupils and parents variables with emotional abuse

**Variables**	**Emotional abuse**				
	**No.**	**%**	**OR**	**(95.0 % CI)**	***P-*value**
**Gender**					
Female*	104	26.1	1.00		
Male	484	72.6	9.94	7.19-13.74	.000
**Family type**					
Nuclear*	388	51.9	1.00		
Extended	200	62.7	1.40	1.02-1.90	.056
**Father education**					
Illiterate*	46	62.2	1.00		
Read/write	162	57.7	.84	.46-1.51	.549
Basic	61	59.2	.73	.50-1.06	.094
Secondary	91	45.0	.99	.60-1.65	.967
University	228	56.2	.58	.39-.86	.007

* Reference category.

## Discussion

This study is the first in Aden to explore the issues related to pupils' reports of emotional abuse in the school context. This study was conducted to describe the prevalence of emotional abuse by teachers and administration staff in schools. In addition, it provides empirical evidence on how pupils and parents socio-demographic characteristics were associated with pupils' victimization by school staff in Aden governorate.

It is often assumed that the consequences of emotional abuse are not as severe as those of more obvious forms of abuse [24], but in fact, relatively little is known about the magnitude of the problem of child emotional abuse worldwide. The prevalence of emotional abuse in the current study was 55.2%; it was relatively lower than that found in Iran 59.9% [17], but higher than that reported in Cyprus 33.1% [15] and Taiwan 11.6% [23]. Prevalence rates of emotional abuse are difficult to ascertain because they capture a wide range of teaching behaviors, and there is little, or no consensus across studies as to what phenomena should be included. The observed differences between countries could be examined from several perspectives. First, it might be that the overall teacher behavior in the various countries is genuinely different, and emotional abuse of pupils was frequently noted as a means of controlling the classroom [25]. Second, emotional abuse might be conceptualized differently in these countries, and what “falls outside the range of acceptability” is variously defined [26]. Third, the methodology used, the study population, and the timing frame are not the same for all studies. Finally, cultural context could differently influence the perception of the abuse acts, where a lot of acts might be culturally accepted ways of disciplining pupils and are therefore not perceived to be abuse acts by a teacher or school administrators [11,27].

In our study, the relatively high frequency of teachers' emotionally abusive behavior could be explained by different social, cultural, and organization factors. For example, some abusive behaviors might be culturally acceptable, such as "shouting and calling names". On the other hand, teachers might not be fully aware about child rights in addition to lack of comprehensive disciplinary rules concerning the appropriate management of pupils.

The current study indicates that males had significantly higher odds of experiencing emotional abuse than females, which is consistence with previous studies [15,17,28]. This could be interpreted by the fact that boys are more likely to have a conflicting relationship with their teachers than their female counterparts [29,30]. Interestingly, inattention seemed to provoke the teacher's scorn, especially for boys but not for girls. One possible explanation may be that, in girls, a lack of attention may be considered a temporary lapse and thus, be more readily excused or ignored than in boys. In line with this notion, low-achieving boys have been shown to have been treated more negatively by teachers than their female counterparts [4]. On the other hand, it has been noticed that male pupils are more involved in disruptive behaviors in the classroom, such as noise, shouting, fighting and throwing up, to get attention compared to girls. This may prompt teachers to emotionally abuse boys more than girls. Moreover, it was concluded that, compliance, following rules and being neat and orderly, is valued and reinforced in many classrooms. In addition, they stressed that these are behaviors, which are typically associated with girls rather than boys, and this may account for the reason why boys experience emotional abuse more often than girls from their teachers [31]. Furthermore, it was reported that poor academic performance of male pupils might explain the higher prevalence of emotional abuse among boys. They found that low achieving boys have been more emotionally abused by both male and female teachers compared to girls [29]. Accordingly, we can conclude that gender is a strong predictor of emotional abuse.

With respect to pupils' age group, the findings indicated that the risk of emotional abuse in schools increase when pupils grow up. This result is consistent to what was found in Iran [17]. Pupils aged 16–17 years are typically those who have failed school years and are thus prone to experience emotional abuse, being threatened with school expulsion [17]. Another explanation is that teachers of older pupils use scolding and grades deduction more [25]. Insulting pupils in our study was 28.4% very much lower than what was reported in an earlier study from Yemen where 42.3% of the pupils aged 6–15 years old claimed that they were insulted at school when they make a mistake [20].

Emotional abuse is prevalent in 7^th^, 8th, and 9^th^ grades; however, there was no statistical significant association with school grade, though findings show relatively higher odds among 8^th^ graders. This could be explained by the increasing conflict situations between teachers and the 8^th^ grade pupils because of their higher claims for autonomy. Furthermore, it is well known that early adolescence is often a time of increased emotional sensitivity, and even relatively benign comments by teachers can at times be interpreted by adolescent pupils as offending [26]. Further studies are required to examine more closely the characteristics of abusive teachers' behaviors in relation to the pupils' school grade and age groups.

For the assessment of the prevalence of emotional abuse, it is also important to analyze how the family structure and parent's socio-demographic characteristics may be related to pupil's abuse in schools. In our study, we examined the relationship between the prevalence of emotional abuse in a school and the educational level, family type, and marital status of the pupil's parents.

The study findings showed that pupils living in extended families have higher odd of reporting being emotionally abused at school. This could be interpreted according to the social learning theory, where pupils from extended families live in crowded homes and witness family violence. In addition, children remain outdoors long hours observing and imitating aggressive behaviors that affects their relationship with teachers and make them at higher risks of emotional abuse [32].

The study findings revealed that pupils whose parents have low education level, exhibited higher odds of emotional abuse but was not statistically significant.

Parents with low education level may be less able to avail themselves of resources for coping with family problems, avoiding abusive relationships, and for that, they solve their problems aggressively [33]. Children of those families usually witness violence at home, which negatively influence their behavior at school and make them at higher risks of abuse. The findings of our study indicate that the higher education level of fathers play a protective role against child abuse at school.

## Conclusion

Emotional abuse of schoolchildren is a highly prevalent problem with social, cultural and health dimensions. Child gender, family type, and father's education level were the main predictors for emotional abuse. Accordingly, appropriate social, legislative and administrative interventions at the family, school and community levels are essential to deal with the problem of schoolchildren emotional abuse by their teachers. Further studies at a national level are required for better understanding the different dimensions of this problem.

## Competing interests

The authors declare that they have no competing interests.

## Authors’ contributions

ASS, carried out the design of the study, collected the data, completed all statistical analyses, interpreting the data, and drafted the manuscript. ASH, has been actively involved in drafting the manuscript and revising it critically for important intellectual content; and has given final approval of the version to be published.

## Pre-publication history

The pre-publication history for this paper can be accessed here:

http://www.biomedcentral.com/1471-2458/12/647/prepub
